# An observational prospective study based on a large cohort of HIV-negative neurosyphilis patients with particular reference to the Jarisch-Herxheimer reaction

**DOI:** 10.1007/s10096-024-04810-1

**Published:** 2024-04-01

**Authors:** Rui-Rui Peng, Juan Wu, Wei Zhao, Lin Zhu, Zhifang Guan, Xin Gu, Mei Shi, Junjun Yu, Yanchun Cheng, Pingyu Zhou

**Affiliations:** Sexually Transmitted Disease Institute, Shanghai Skin Disease Hospital, Tongji University School of Medicine, 1278 Baode Road, Shanghai, 200443 China

**Keywords:** Neurosyphilis, Jarisch-herxheimer reaction, Severe reaction, Epidemiology, Clinical spectrum

## Abstract

**Purpose:**

The purpose of this study is to outline a complete picture of Jarisch-Herxheimer reaction (JHR) in the central nervous system among HIV-negative neurosyphilis patients.

**Methods:**

A prospective study cohort of 772 cases with almost all stages of neurosyphilis depicted the features of JHR including occurrence rate, risk profiles, clinical manifestations, medical management and prognosis.

**Results:**

The total occurrence rate of JHR was 9.3% (95% CI, 7.3-11.4%), including 4.1% (95% CI, 2.7-5.6%) with severe JHR. The reaction started 5 h after treatment initiation, peaked after 8 h, and subsided after 18 h. Patients with severe JHR experienced a longer recovery time (26 h). Patients with general paresis (OR = 6.825), ocular syphilis (OR = 3.974), pleocytosis (OR = 2.426), or a high CSF-VDRL titre (per log_2_ titre increase, OR = 2.235) were more likely to experience JHR. Patients with general paresis had an 11.759-fold increased risk of severe JHR. Worsening symptoms included cognitive impairment, mania, nonsense speech, and dysphoria, while symptoms of hallucination, urination disorder, seizures, myoclonus, or aphasia appeared as new-onset symptoms. Neurosyphilis treatment did not need to be interrupted in most patients with JHR and could be reinstated in patients with seizures under supportive medication when JHR subsided.

**Conclusion:**

Severe JHR displayed a 4.1% occurrence rate and clinicians should pay particular attention to patients at a higher risk of JHR. The neurosyphilis treatment regime can be restarted under intensive observation for patients with severe JHR and, if necessary, supportive medication should be initiated and continued until the end of therapy.

## Introduction

The Jarisch-Herxheimer reaction (JHR) is a febrile transient inflammatory syndrome triggered hours after antibiotic treatment of spirochete infections, including syphilis [1, 2]. Clinically, JHR is typically manifested as an abrupt onset of constitutional symptoms, including fever, chills, malaise, headache, tachypnoea, tachycardia, nausea, myalgias, arthralgias, and exacerbation of existing cutaneous lesions, which can subside spontaneously among patients with early syphilis [3–5]. However, neurosyphilis patients may undergo significant aggravation of neuropsychiatric symptoms due to a severe JHR in the central nervous system (CNS), including stroke, seizures, and alterations in consciousness which could be life-threatening [6–9].

The world has experienced a resurgence of syphilis since the beginning of the 21st century [10, 11] Syphilis has also returned to China with a vengeance [12]. It has been estimated that 34 cases per 100,000 persons were diagnosed as emerging infectious syphilis in 2021 in China [13], and the epidemiology of neurosyphilis has largely paralleled that of active syphilis [14]. Neurosyphilis can occur during any stage of *Treponemal pallidum* (*TP*) infection because *TP*, the causative agent of syphilis, can invade the CNS within days after exposure [15]. Therefore, prompt antimicrobial treatment is critical to alleviating the intensification of clinical conditions [16–18]. Consequently, JHR will inevitably occur during the treatment of neurosyphilis.

The detailed epidemiology and clinical information of JHR in neurosyphilis patients has not been well described due to the paucity of population-based data. Here we depict the complete picture of JHR, including occurrence rate, risk profiles, clinical features, medical management, and prognosis, among 772 HIV-negative neurosyphilis patients in Shanghai, China.

## Materials and methods

### Ethics statement

This observational prospective study was carried out at the Sexually Transmitted Disease Institute of Shanghai Skin Disease Hospital from July 2017 to December 2020. It was approved by the medical ethics committee of Shanghai Skin Disease Hospital (ethics number: 2016-011) and conducted according to the principles expressed in the Declaration of Helsinki. The written informed consent was obtained from all enrolled cases and all data for research analyses were anonymized.

### Case definition and patient recruitment

Neurosyphilis was defined as having (1) serologic evidence of syphilis, (2) a reactive cerebrospinal fluid-venereal disease research laboratory (CSF-VDRL) test and CSF-*Treponema pallidum* particle assay (CSF-TPPA), or nonreactive CSF-VDRL but reactive CSF-TPPA with (3) elevated CSF-protein levels (> 500 mg/L) and/or pleocytosis (> 10 white blood cells/µL) in the absence of other known causes of these abnormalities [16]. Symptomatic neurosyphilis was defined as having CSF abnormalities the above mentioned in the presence of neuropsychiatric signs or symptoms, otherwise it was defined as asymptomatic neurosyphilis [16]. The criteria of each type of symptomatic neurosyphilis in detail were classified according to the STD guidelines [16–18].

JHR was defined as having constitutional symptoms after initiation of a neurosyphilis regimen without other known causes, including fever, chills, headache, malaise, some neuropsychiatric symptoms etc. [2]. It was further categorized into two subtypes according to the severity of clinical presentations [2, 19, 20] as follows: (1) an elevation in body temperature (≥ 38.0℃) accompanied by flu-like symptoms with no neuropsychiatric symptoms flare in symptomatic neurosyphilis or no emergence of new neuropsychiatric symptoms in asymptomatic neurosyphilis, hereinafter referred to as a mild JHR; (2) an exacerbation of existing neuropsychiatric symptoms, or emergence of new neuropsychiatric symptoms regardless of an increase in body temperature, hereinafter referred to as a severe JHR.

Neurosyphilis patients were recruited if they (1) were above 18 years old; (2) underwent a first episode of neurosyphilis treatment; (3) did not receive oral or intravenous or intramuscular usage of antibiotics in the past 3 months; (4) did not have a recent history of other infections (e.g.: viral, bacterial, protozoal); (5) denied a history of febrile diseases in the past 3 months; (6) denied a history of neurologic disorders and psychosis; (7) had a negative status of HIV. First-line therapy of neurosyphilis was aqueous crystalline penicillin G, 4 million units given intravenously every 4 h for 14 days, or 1 g of ceftriaxone administered intravenously every 12 h for 14 days in case of allergy to penicillin [16–18].

### Collection of clinical data

All patients were hospitalized for neurosyphilis treatment. The baseline data were recorded as follows: gender, age, neurosyphilis type, accompanying features caused by syphilis, history of syphilis treatment, current neurosyphilis therapy regimen, serum titre of toluidine red unheated serum test (TRUST), the CSF examination results, including WBC count, protein content, and VDRL titre (BD BBL™ VDRL Antigen, REF 240,764, Franklin Lakes, NJ, USA).

At the beginning of neurosyphilis treatment, clinical monitoring was done by the study nurse and doctor at regular intervals, namely every 2 h for the first 12 h, then every 4 h until the remission of JHR. The following detailed data were recorded: axillary temperature, blood pressure, heart rate, respiratory rate (by the nurse), and the clinical features during JHR such as changes in cognition, mental status, consciousness, gait, and movement (by the doctor). Complete blood count and blood culture were carried out in all febrile patients to rule out other potential infections.

### Statistical analysis

All data were recorded on a specially designed chart for each patient and independently double-coded with Epidata software (version 3.1; Denmark). Data were analysed using IBM SPSS statistics (version 24.0; Chicago, IL, USA). Descriptive statistics was used to calculate the median, interquartile range (IQR), percentage, frequency, and 95% confidence interval (CI). The titres of serum TRUST and CSF-VDRL were normalized by log_2_ transformation. A chi-square test (*p* < 0.05 indicating statistical significance) was applied to analysed potential factors associated with JHR. A multivariate binary logistic regression model was established to further identify factors that were independently associated with JHR if significant factors were found by chi-square test.

## Results

### Baseline characteristics of neurosyphilis patients with or without JHR

**Fig. 1:**
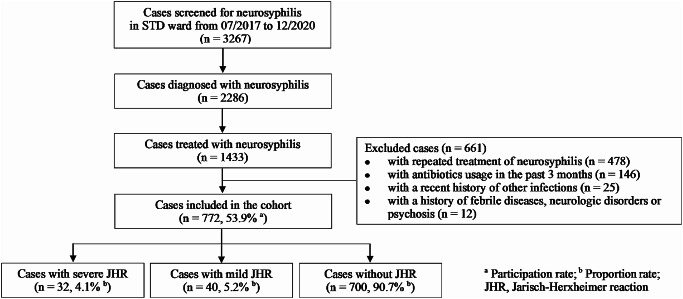
Enrolment flow chart of neurosyphilis cases

From July 2017 to December 2020, 772 neurosyphilis patients were recruited according to the inclusion criteria of the study (Fig. 1). The majority of patients (75.8%) were male. The median age was 59 years (IQR, 53 to 65). The predominant types of neurosyphilis were asymptomatic neurosyphilis (337 cases, 43.7%) and general paresis (GP, 299 cases, 38.7%), followed by meningovascular syphilis (77 cases, 10.0%), tabes dorsalis (TD, 71 cases, 9.2%), syphilitic meningitis (11 cases, 1.4%) and intracranial gumma (3 cases, 0.4%). Simultaneously, 51 cases were complicated with ocular syphilis, 9 with syphilitic skin lesions, 6 with secondary epilepsy caused by neurosyphilis, 3 with cardiovascular syphilis, and 1 with otosyphilis. Furthermore, 49.4% of patients received syphilis treatment at least 3 months ago prior to enrolment in this study. The median titre of TRUST was 1:32 (IQR, 1:16 to 1:64). CSF-VDRL was positive in 670 cases (670/772, 86.8%) and the median titre of VDRL was 1:4 (IQR, 1:1 to 1:8). Additionally, 54.8% of patients had a CSF-WBC count greater than 10 cells/uL (median, 14; IQR, 4 to 46) and 84.7% had a CSF-protein concentration higher than 500 mg/L (median, 792; IQR, 580 to 1055) (Table 1).

**Table 1:** Characteristics of neurosyphilis patients included in the current study

Factors	No. Patients (%) ^a^ (*N* = 772)	No. Patients with JHR (%) ^a^ (*N* = 72)	P value in multivariate regression ^b^
Male	585 (75.8)	52 (72.2)	0.105
Age (yr.), median (IQR)	59 (53–65)	57 (52–62)	0.158
Types of neurosyphilis ^c^			
Asymptomatic neurosyphilis	337 (43.7)	6 (8.3)	0.212
** General paresis**	299 (38.7)	59 (81.9)	< 0.001
Meningovascular syphilis	77 (10.0)	6 (8.3)	0.234
Tabes dorsalis	71 (9.2)	3 (4.2)	0.167
Syphilitic meningitis	11 (1.4)	2 (2.8)	
Intracranial gumma	3 (0.4)	0 (0)	
Accompanying features caused by syphilis			
** Ocular syphilis**	51 (6.6)	4 (5.6)	0.029
Syphilitic skin lesions	9 (1.2)	1 (1.4)	
Secondary epilepsy	6 (0.8)	0 (0)	
Cardiovascular syphilis	3 (0.4)	0 (0)	
Otosyphilis	1 (0.1)	0 (0)	
**Treatment of syphilis before** ^d^	381 (49.4)	5 (6.9)	< 0.001
TRUST titre, median (IQR)	1:32 (1:16 − 1:64)	1:32 (1:16 − 1:64)	0.291
Cerebrospinal fluid			
** VDRL titre**, median (IQR)	1:4 (1:1–1:8)	1:8 (1:4 − 1:16)	0.038
** WBC count** (cells/µL), median (IQR)	14 (4–46)	48 (16–70)	0.022
Protein concentration (mg/L), median (IQR)	792 (580–1055)	1168 (835–1532)	0.231
Neurosyphilis regimen			0.329
Aqueous crystalline penicillin G	679 (88.0)	62 (86.1)	
Ceftriaxone	93 (12.0)	10 (13.9)	

^a^ Data are number of patients (percentage), unless otherwise indicated. ^b^ Nonsignificant factors based on the univariate analysis were not included in the multivariate regression model. Significant factors associated with JHR in the multivariate regression model are shown in bold. ^c^ Total number is greater than 772 because of multiple diagnoses for some patients. ^d^ Patients have received benzathine penicillin for syphilis before, but not for neurosyphilis, which do not reach treponemacidal levels in the central nervous system. Abbreviations: JHR, Jarisch-Herxheimer reaction; IQR, interquartile range; TRUST, toluidine red unheated serum test; VDRL, venereal disease research laboratory test; WBC, white blood cell

A total of 72 patients displayed JHR following the first dose of the neurosyphilis regimen. Of these, 72.2% were male, 93.1% had no history of syphilis treatment, the median age was 57 years (IQR, 52 to 62) and 59 cases were diagnosed with GP (81.9%). In addition, 6 cases were diagnosed with asymptomatic neurosyphilis, another 6 cases were diagnosed with meningovascular syphilis, 3 cases with TD, and 2 cases with syphilitic meningitis. In patients suffering from ocular syphilis and syphilitic skin lesions, 4 and 1 cases, respectively, experienced JHR. The median titre of TRUST was 1:32 (IQR, 1:16 to 1:64). The median titre of CSF-VDRL was 1:8 (IQR, 1:4 to 1:16). Furthermore, 86.1% of cases had a CSF-WBC count greater than 10 cells/uL (median, 48; IQR, 16 to 70) and 98.6% had a CSF-protein concentration greater than 500 mg/L (median, 1168; IQR, 835 to 1532). (Table 1)

### The occurrence rate and risk profiles of JHR in neurosyphilis patients

The total occurrence rate of JHR was 9.3% (72/772; 95% CI, 7.3–11.4%) among 772 neurosyphilis patients. Specifically, 5.2% (40/772, 95% CI, 3.6–6.7%) of patients suffered a mild JHR while 4.1% (32/772, 95% CI, 2.7–5.6%) suffered a severe JHR. The occurrence rates of JHR in different types of neurosyphilis were as follows: 19.7% (59/299) in GP, 18.2% (2/11) in syphilitic meningitis, 7.8% (6/77) in meningovascular syphilis, 4.2% (3/71) in TD, and 1.8% (6/337) in asymptomatic neurosyphilis. Particularly, the occurrence rate of JHR was 7.8% (4/51) in neurosyphilis patients who also had ocular syphilis.

A multivariate logistic regression model showed that previous treatment of syphilis was related to a lower risk of JHR (OR, 0.126; 95% CI, 0.048 to 0.332; *p* < 0.001). Conversely, a neurosyphilis patient had a greater risk of JHR if diagnosed with GP (OR, 6.825; 95% CI, 3.200 to 14.558; *p* < 0.001), suffered from ocular syphilis (OR, 3.974; 95% CI, 1.156 to 13.665; *p* = 0.029), had pleocytosis in CSF (OR, 2.426; 95% CI, 1.139 to 5.166; *p* = 0.022), or had a higher CSF-VDRL titre (per log_2_ titre increase, OR, 2.235; 95% CI, 1.047 to 4.772; *p* = 0.038). Meanwhile, a patient with GP was more likely to suffer from severe JHR (OR, 11.759; 95% CI, 1.427 to 96.872; *p* = 0.022). Gender, age, TRUST titre in serum, protein concentration in CSF, and neurosyphilis regimen had no statistically significant association with JHR.

### Clinical features of JHR in neurosyphilis patients

Overall, the mean onset of JHR occurred at 5 h (range, 0.5 to 13 h), the peak activity was reached at 8 h (range, 0.5 to 20 h), and subsided at 18 h (range, 9 to 30 h) following the first dose of neurosyphilis treatment (Table 2). Patients with a severe JHR experienced a significantly longer recovery time compared with those with a mild JHR (15 h vs. 26 h; *p* < 0.05).

**Table 2:** Effect of JHR on the symptoms of neurosyphilis

Characteristics	No. Patients (%) ^a^ (*N* = 72)
Temp. (℃) in patients with fever ^b^	
Range	38.1–40.4
Median (IQR)	38.8 (38.5–39.3)
Time interval of JHR (hr.) (mean, range)	
Onset	5 (0.5–13)
Peak	8 (0.5–20)
Offset	18 (9–30)
Neuropsychiatric symptoms during JHR	
No symptoms in central nervous system	4 (5.6)
Maintenance of existing symptoms	36 (50.0)
Exacerbation of existing symptoms	17 (23.6)
Occurrence of new symptoms	15 (20.8)
Type of JHR	
Mild JHR ^c^	40 (55.6)
Severe JHR ^d^	32 (44.4)

^a^ Data are number of patients (percentage), unless otherwise indicated. ^b^ Almost all patients had a fever except for one patient. ^c^ elevation in body temperature accompanied by flu-like symptoms without neuropsychiatric symptoms flare. ^d^ exacerbation of existing psychoses and focal neurologic signs, or emergence of new neuropsychiatric symptoms. Abbreviations: JHR, Jarisch-Herxheimer reaction; IQR, interquartile range

**Fig. 2:**
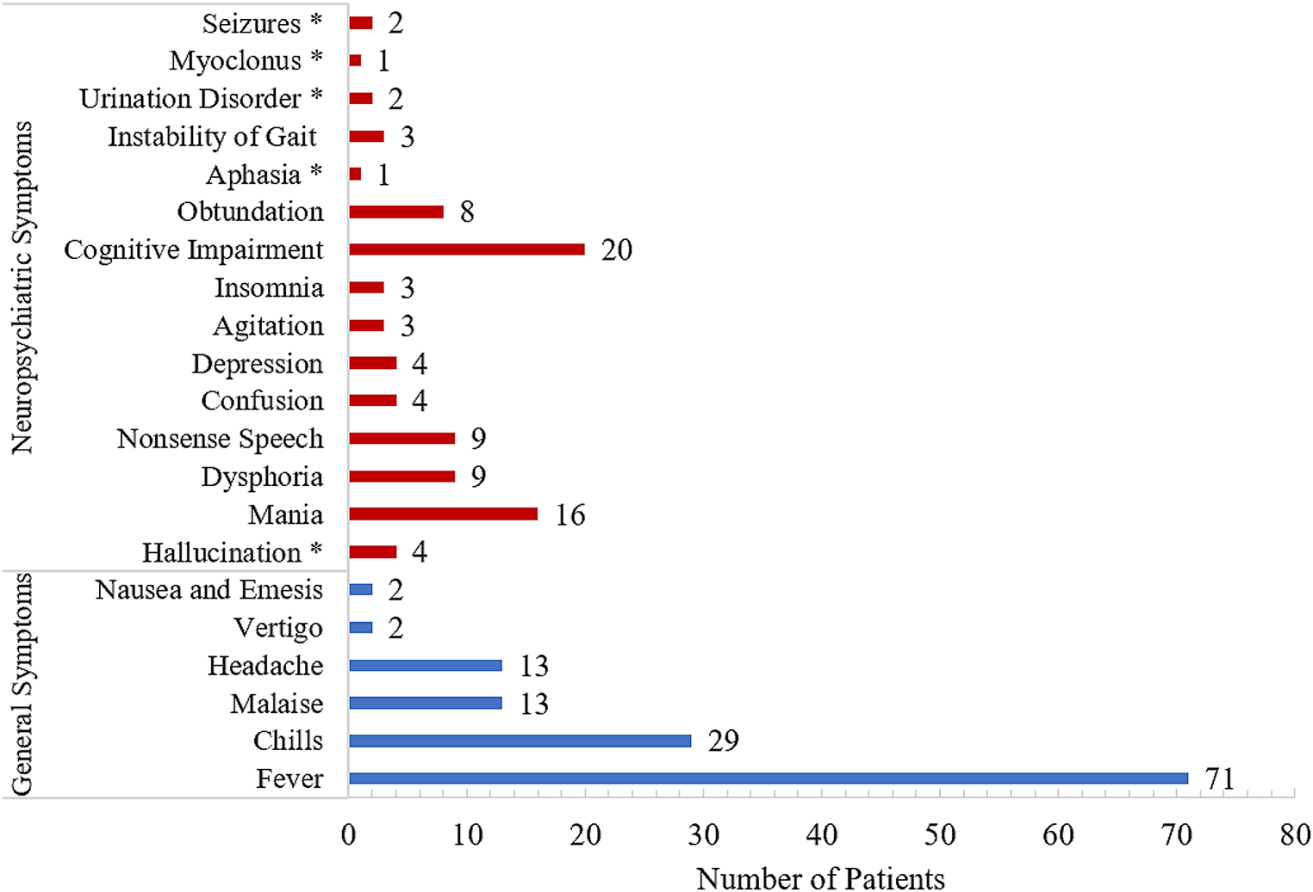
Symptoms occurring during JHR in 72 neurosyphilis patients ^*^ New-onset symptoms during JHR; Arabic numerals indicated the number of patients; *Abbreviations* JHR, Jarisch-Herxheimer reaction

The general symptoms of JHR included fever, chills, malaise, headache, vertigo, nausea and emesis. Most (71 of 72) cases with JHR displayed a fever from 38.1℃ to 40.4℃ with a median of 38.8℃. All febrile patients had negative results on blood culture. A transient and reversible lymphopenia and leucocytosis was found on complete blood count during JHR, but no association was found between the severity of JHR and changes in complete blood count or degree of body temperature elevation. The most frequent neuropsychiatric symptoms were cognitive impairment and mania, followed by nonsense speech, dysphoria, obtundation, hallucination, depression, confusion, agitation, insomnia, instability of gait, urination disorder, seizures, myoclonus, and aphasia. (Fig. 2)

**Fig. 3:**
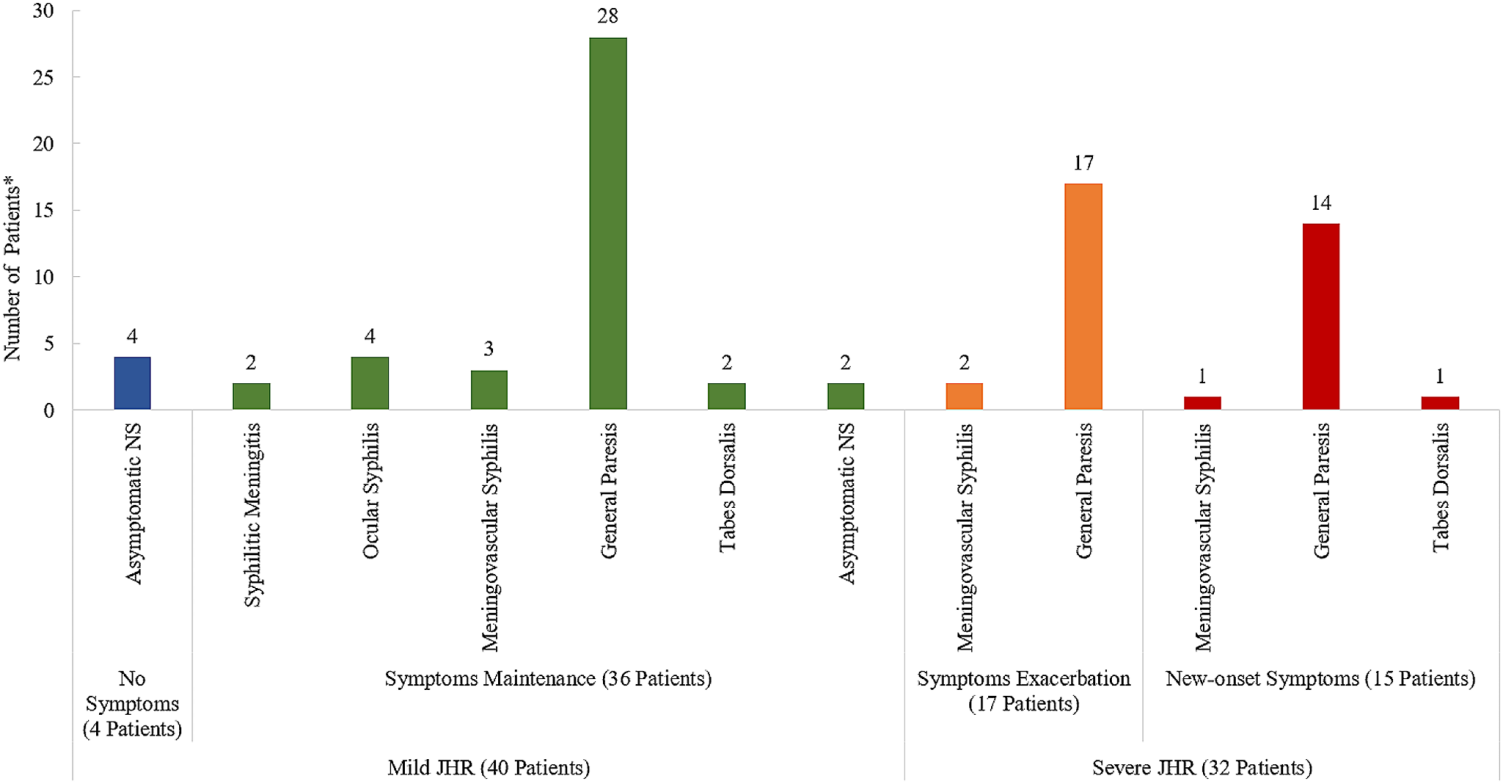
Patient distribution according to neuropsychiatric symptoms during JHR ^*^ Some patients showed a combination of multiple forms of neurosyphilis pathology; Arabic numerals indicated the number of patients; Mild JHR included no neuropsychiatric symptoms and neuropsychiatric symptoms maintenance during JHR; Severe JHR included neuropsychiatric symptoms flare and new neuropsychiatric symptoms during JHR; *Abbreviations* JHR, Jarisch-Herxheimer reaction; NS, neurosyphilis

The predominant diagnosis in patients with JHR was GP, especially in those with a severe JHR. It was observed that 31 patients with GP suffered from a severe JHR, including 17 with a flare-up of existing neuropsychiatric symptoms and 14 who developed new neuropsychiatric symptoms. None of these patients had a history of syphilis treatment. The worsening symptoms mainly included cognitive impairment, mania, nonsense speech, and dysphoria. Hallucination, urination disorder, seizures, myoclonus, or aphasia appeared as new-onset symptoms during JHR. Meanwhile, in 40 patients with a mild JHR, 4 asymptomatic neurosyphilis cases had flu-like symptoms without CNS involvement, while the other 36 cases had no aggravation of existing neuropsychiatric symptoms. (Table 2; Fig. 3)

### The management and prognosis of JHR in neurosyphilis patients

All symptomatic neurosyphilis patients were managed stably with supportive medications before neurosyphilis treatment. Premedication with corticosteroids was not a routine in our cohort except in neurosyphilis patients complicated with ocular syphilis. Antipyretics were used to relieve febrile symptoms when the body temperature was greater than 38.5℃. To mitigate worsening psychosis in patients with GP during a severe JHR, the scheduled doses of olanzapine were increased with the addition of clonazepam. Neurosyphilis treatment was not interrupted in patients with a mild JHR nor was treatment interrupted in most patients with a severe JHR.

One patient with GP had two unremitting generalized seizures for three minutes, 10 min apart, which were triggered 30 min after aqueous crystalline penicillin G infusion. The symptoms gradually subsided 30 h later with the supportive infusion of dexamethasone, diazepam, and mannitol. Subsequently, this patient developed a consecutive episode of obtundation and depression. Aqueous crystalline penicillin G was then reinstituted uneventfully for 14 days under intensive observation. Another patient with GP undergoing complex partial seizures during JHR received aqueous crystalline penicillin G infusion without interruption. This patient did not experience deterioration of her neurologic disorder due to prompt usage of intravenous dexamethasone and diazepam at the instant of JHR occurrence. Only one patient with GP discontinued neurosyphilis treatment and was transferred to a psychiatric ward because of uncontrolled aggressive behaviours. None of the patients with ocular syphilis experienced deterioration of visual acuity during JHR. The neurosyphilis patients complicated with cardiovascular syphilis, gumma, or otosyphilis in this study did not experience JHR during neurosyphilis treatment.

## Discussion

The initial description of JHR in the literature was independently given in 1895 by Adolf Jarisch (an Austrian dermatologist) and in 1902 by Karl Herxheimer (a German dermatologist), who described it as a transient exacerbation in the appearance of mucocutaneous lesions after treating syphilis with mercury [21, 22]. For several decades, JHR was usually defined as a self-limiting condition in early syphilis [21]. Until the 1940 and 1950 s, studies showed that the occurrence rate of JHR varied from 64 to 79% among neurosyphilis patients [19, 23, 24] and that between 1.7 and 11% of patients underwent a severe reaction in the CNS [19]. Since the start of the 21st century, a wide range of occurrence rates (from 8 to 75%) has been reported in JHR among neurosyphilis patients [8, 25, 26].

In the past 10 years, two observational studies have discussed JHR in syphilis patients based on large sample sizes. One study from Taiwan, China, found an overall rate of JHR of 31.5% in 355 syphilis patients, including 34.6% in HIV-infected patients and 25.2% in noninfected patients [3]. The other study from Beijing, China, revealed that the JHR occurred much less frequently with a rate of 1.4% in 1125 syphilis patients [4]. However, the above studies did not investigate neurosyphilis patients. Here we conducted a prospective study among a cohort of 772 neurosyphilis patients with different clinical stages, including asymptomatic, early, and late parenchymatous neurosyphilis. We found a total occurrence rate of JHR of 9.3% among HIV-negative neurosyphilis patients, including 5.2% in a mild JHR group and 4.1% in a severe JHR group.

The lower occurrence rate of JHR in neurosyphilis patients in our study compared with those in previous studies in the 1940 and 1950 s (9.3% vs. 64–79%) [19, 23, 24] probably resulted from the current extensive usage of antibiotics. Particularly, penicillin- or cephalosporin-related antibiotics used for infectious diseases had a partial effect on syphilis by reducing the loads of *TP* in the body. The occurrence of JHR might depend on the spirochete loads in the body [3, 27]. Our finding that previous syphilis treatment was a protective factor (0.126-fold risk) could verify this viewpoint. However, interestingly, when considering the severity of JHR, the rate of 4.1% in the severe reaction group was consistent with previous studies (1.7–11.0%) [19]. Additionally, almost all patients with severe JHR in this study were diagnosed with GP and denied a history of syphilis treatment. Therefore, clinicians should be particularly vigilant with these patients after initiation of the neurosyphilis treatment regime.

Previous studies listed several clinical manifestations of JHR in the CNS including hallucination, changes in consciousness or orientation, seizures, hemiparesis, facial nerve weakness, diplopia, stroke, and abnormal magnetic resonance imaging or electroencephalography [7–9, 20, 25, 28–32]. Our study found that in addition to the above symptoms, negative symptoms of psychoses such as depression, agitation, or insomnia could be the main manifestations, which were not mentioned before. Moreover, symptoms including aphasia, urination disorder, instability of gait, or myoclonus also could be presented during JHR in neurosyphilis patients. The JHR in the CNS was more likely in neurosyphilis patients who were diagnosed with GP (6.825-fold risk), were complicated with ocular syphilis (3.974-fold risk), had a pleocytosis (2.426-fold risk) and a higher CSF-VDRL titre (per log_2_ titre increase, 2.235-fold risk). Particularly, patients with GP had an 11.759-fold increased risk of severe JHR compared with other neurosyphilis patients.

Administration of corticosteroids prior to antibiotic treatment has been recommended to prevent JHR [16, 18]. Although corticosteroids can attenuate febrile reaction in patients with early-stage syphilis, a possible disadvantage of their use may be the inhibition of phagocytosis which is useful for clearing *TP* and for preventing syphilis relapses [33–35]. Corticosteroids were also considered to be of no benefit in the management of symptomatic neurologic complications during JHR [20, 36]. We did not prescribe corticosteroids as a routine medication except for ocular syphilis patients and two patients with seizures during JHR. In our study, almost all neuropsychiatric symptoms, including flare-ups of symptoms and new-onset symptoms during JHR, subsided no later than 30 h following treatment initiation. When JHR subsided, the neurosyphilis therapy could be reinstated, even in patients with seizures, under intensive observation and supportive medication.

Worldwide, syphilis is far from eradicated, especially in resource-limited areas, and it can affect any part of the neuraxis at any stage of infection [10, 15, 37]. There is a growing consensus that neurosyphilis patients can benefit from prompt standardized treatment [38]. The current study is an observational prospective study outlining the JHR rates in a cohort of 772 patients diagnosed with almost all stages of HIV-negative neurosyphilis. All patients were admitted to the STD ward and clinical data were collected at regular intervals in order to reduce the unrecognized bias and recalling bias. However, there were some limitations to this study. Firstly, we could not further investigate the imaging characteristics of patients with neuropsychiatric symptoms during JHR because electroencephalography and brain MRI were unavailable. Secondly, the present study did not test for potential biomarkers such as cytokines in the CSF during JHR. And then, the definitions for JHR were nonspecific, which might be limited by the absence of readily available surrogate markers to detect JHR. Finally, neurosyphilis patients that were co-infected with HIV were not included in the cohort due to the paucity of HIV-positive cases. These factors might limit the generalization of our findings.

## Conclusion

The current study describes the largest cohort of HIV-negative neurosyphilis patients in the modern era. The total occurrence rate of JHR was 9.3% and that of severe JHR was 4.1%. It is recommended that the clinician should be forewarned and pay great attention to those patients who are diagnosed with GP or ocular syphilis, received no syphilis care previously, or displayed pleocytosis or a high CSF-VDRL titre. The treatment of neurosyphilis can be reinitiated in patients with a severe JHR under supportive medication following the subsiding of JHR.

## Data Availability

Data that support the findings of this study are available from the corresponding author on reasonable request.

## References

[1] Butler T (2017). The Jarisch-Herxheimer reaction after antibiotic treatment of Spirochetal infections: a review of recent cases and our understanding of Pathogenesis. Am J Trop Med Hyg.

[2] Dhakal A, Sbar E (2022) Jarisch Herxheimer reaction. StatPearls. Treasure Island (FL): StatPearls Publishing Copyright © 2022. StatPearls Publishing LLC 32491752

[3] Yang CJ, Lee NY, Lin YH (2010). Jarisch-Herxheimer reaction after penicillin therapy among patients with syphilis in the era of the hiv infection epidemic: incidence and risk factors. Clin Infect Dis.

[4] Li J, Wang LN, Zheng HY (2013). Jarisch-Herxheimer reaction among syphilis patients in China. J Eur Acad Dermatol Venereol.

[5] Arando M, Fernandez-Naval C, Mota-Foix M (2018). The Jarisch-Herxheimer reaction in syphilis: could molecular typing help to understand it better?. J Eur Acad Dermatol Venereol.

[6] Belum GR, Belum VR, Chaitanya Arudra SK, Reddy BS (2013). The Jarisch-Herxheimer reaction: revisited. Travel Med Infect Dis.

[7] Kobayashi J, Nakagawa Y, Tobisawa S, Isozaki E, Koide R (2011). Deterioration of MRI findings related to Jarisch-Herxheimer reaction in a patient with neurosyphilis. J Neurol.

[8] Punia V, Rayi A, Sivaraju A (2014) Stroke after Initiating IV Penicillin for Neurosyphilis: A Possible Jarisch-Herxheimer Reaction. Case reports in neurological medicine 2014: 548179 10.1155/2014/548179 PMC4238263 25431710

[9] Rissardo JP, Caprara ALF, Silveira JOF (2019). Generalized convulsive Status Epilepticus secondary to Jarisch-Herxheimer reaction in Neurosyphilis: a Case Report and Literature Review. Neurologist.

[10] Ghanem KG, Ram S, Rice PA (2020). The modern epidemic of Syphilis. N Engl J Med.

[11] Peeling RW, Mabey D, Kamb ML, Chen XS, Radolf JD, Benzaken AS (2017). Syphilis. Nat Rev Dis Primers.

[12] Chen ZQ, Zhang GC, Gong XD (2007). Syphilis in China: results of a national surveillance programme. Lancet.

[13] Overview of notifiable infectious diseases in China in 2021 (2023) Available at: http://www.nhc.gov.cn/jkj/s3578/202204/4fd88a291d914abf8f7a91f6333567e1.shtml Accessed 7 May 2023

[14] Shi M, Peng RR, Gao Z (2016). Risk profiles of neurosyphilis in HIV-negative patients with primary, secondary and latent syphilis: implications for clinical intervention. J Eur Acad Dermatol Venereol.

[15] Lafond RE, Lukehart SA (2006). Biological basis for syphilis. Clin Microbiol Rev.

[16] National Center for STD Control (2020). Guidelines for diagnosis and treatment of syphilis, gonorrhea and genital Chlamydia trachomatis infection (2020). Chin J Dermatol.

[17] Workowski KA, Bachmann LH, Chan PA (2021). Sexually transmitted infections Treatment guidelines, 2021. MMWR Recomm Rep.

[18] Janier M, Unemo M, Dupin N, Tiplica GS, Potočnik M, Patel R (2021). 2020 European guideline on the management of syphilis. J Eur Acad Dermatol Venereol.

[19] Hoekenga MT, Farmer TW (1948). Jarisch-Herxheimer reaction in neurosyphilis treated with penicillin. Arch Intern Med.

[20] Zifko U, Lindner K, Wimberger D, Volc B, Grisold W (1994). Jarisch-Herxheimer reaction in a patient with neurosyphilis. J Neurol Neurosurg Psychiatry.

[21] Herxheimer K, Krause D (1902). Ueber Eine Bei Syphilitischen Vorkommende quecksbilberreaktion. Dtsch MedWochenschr.

[22] Jarisch A (1895). Therapeutische Versuche Bei Syphilis. Wien MedWochenschr.

[23] Scott V, Maxwell RW, Skinner JS (1949). The Jarisch-Herxheimer phenomen in late syphilis. JAMA.

[24] Heyman A, Sheldon WH, Evans LD (1952). Pathogenesis of the Jarisch-Herxheimer reaction; a review of clinical and experimental observations. Br J Vener Dis.

[25] Silberstein P, Lawrence R, Pryor D, Shnier R (2002). A case of neurosyphilis with a florid Jarisch-Herxheimer reaction. J Clin Neurosci.

[26] Davis LE, Oyer R, Beckham JD, Tyler KL (2013). Elevated CSF cytokines in the Jarisch-Herxheimer reaction of general paresis. JAMA Neurol.

[27] Breuer A, Megged O, Kashat L, Assous MV (2021). Quantitative real-time PCR in Borrelia persica tick-borne relapsing fever demonstrates correlation with the Jarisch-Herxheimer reaction. Eur J Clin Microbiol Infect Dis.

[28] Hagiya H, Deguchi K, Kawada K, Otsuka F (2015). Neurosyphilis is a long-forgotten disease but still a possible etiology for Dementia. Intern Med (Tokyo Japan).

[29] Kojan S, Van Ness PC, Diaz-Arrastia R (2000). Nonconvulsive status epilepticus resulting from Jarisch-Herxheimer reaction in a patient with neurosyphilis. Clin EEG (Electroencephalography).

[30] Gurses C, Kurtuncu M, Jirsch J (2007). Neurosyphilis presenting with status epilepticus. Epileptic Disorders: Int Epilepsy J Videotape.

[31] Bucher JB, Golden MR, Heald AE, Marra CM (2011). Stroke in a patient with human immunodeficiency virus and syphilis treated with penicillin and antiretroviral therapy. Sex Transm Dis.

[32] Zhang SQ, Wan B, Ma XL, Zheng HM (2008). Worsened MRI findings during the early period of treatment with penicillin in a patient with general paresis. J Neuroimaging.

[33] Sheldon WH, Heyman A (1949). Morphologic changes in syphilitic lesions during the Jarisch-Herxheimer reaction. Am J Syph Gonorrhea Vener Dis.

[34] Gudjonsson H, Skog E (1968). The effect of prednisolone on the Jarisch-Herxheimer reaction. Acta Derm Venereol.

[35] Zhou PY, Liao KH, Xu M, Wang X (2001). Influence of corticosteroid on peripheral blood lymphocyte in secondary Syphilis and Prognosis of Syphilis. Chin J Dermatol.

[36] Fekade D, Knox K, Hussein K (1996). Prevention of Jarisch-Herxheimer reactions by treatment with antibodies against tumor necrosis factor alpha. N Engl J Med.

[37] Ropper AH (2019). Neurosyphilis. N Engl J Med.

[38] Musher DM (2008). Neurosyphilis: diagnosis and response to treatment. Clin Infect Dis.

